# Ablation of CFRP Modified with Copper and Calcium Hydroxyapatites by Femtosecond Laser Pulses for Further Material Cutting and Milling Applications

**DOI:** 10.3390/polym18111284

**Published:** 2026-05-23

**Authors:** Paulius Šlevas, Orestas Ulčinas, Sergej Orlov, Egidijus Vanagas, Anna Bilousova, Denys Baklan, Oleksiy Myronyuk

**Affiliations:** 1 Coherent Optics Laboratory, Optoelectronics Department, Center for Physical Sciences and Technology, Sauletekio Ave. 3, LT-10257 Vilnius, Lithuania; paulius.slevas@ftmc.lt (P.Š.); orestas.ulcinas@wophotonics.com (O.U.);; 2 Department of Chemical Technology of Composite Materials, Chemical Technology Faculty, Igor Sikorsky Kyiv Polytechnic Institute, Beresteiskyi Ave. 37, 03056 Kyiv, Ukraine; a.bilousova@kpi.ua (A.B.); d.baklan@kpi.ua (D.B.); o.myronyuk@kpi.ua (O.M.)

**Keywords:** CFRP, carbon fiber, copper and calcium hydroxyphosphates, laser processing, cutting, ablation

## Abstract

The interaction of femtosecond laser ultrashort pulses with carbon fiber-reinforced polymer (CFRP) based on epoxy resin modified with different ratios of copper hydroxyapatite (Cu-HAp) and calcium hydroxyapatite (Ca-HAp) was investigated. Ablation efficiency was examined for two CFRP groups containing 1 wt% and 5 wt% Cu-HAp in the epoxy matrix, and in both cases, the maximum ablation efficiency was obtained at a fluence of about 6.4–7.5 $J/cm^{2}$. The corresponding energy-specific volumes were slightly higher for 1 wt% Cu-HAp (6.95 μm^3^/μJ) and lower for 5 wt% Cu-HAp (6.26 μm^3^/μJ), and at higher fluence, the ablation efficiency decreased smoothly, indicating a limited optimum fluence window for a given CFRP composition. A similar behaviour was observed for epoxy compounds containing 5 wt% total hydroxyapatite, both for Cu-HAp:Ca-HAp = 75:25 and 50:50 mixtures, which showed nearly identical maxima of energy-specific volume around 6.06 μm^3^/μJ at 6.4 $J/cm^{2}$. Epoxy resin without carbon fibers, loaded with 1 wt% and 5 wt% Cu-HAp, exhibited higher energy-specific volumes of about 9–10 μm^3^/μJ and 9–13 μm^3^/μJ, respectively, at around 10 $J/cm^{2}$, followed by a decay of ablation efficiency at higher fluence. Finally, cutting and milling experiments on CFRP demonstrated acceptable surface quality and processing rates under femtosecond laser irradiation, confirming realistic prospects for advanced CFRP fabrication using optimized ablation conditions.

## 1. Introduction

Carbon fiber-reinforced polymer (CFRP) is used in a wide and diverse range of applications [1], from load-bearing frames and structural components in the automotive, marine and aerospace industries to more domestic applications in construction, furniture and sports equipment [2,3]. The outstanding properties of CFRP-based products include high specific strength and stiffness, where the strength of carbon fiber-based structures is comparable to that of steel while the low density of the composite provides weight savings that can exceed the critical specifications of steel-based products [4,5,6]. By tailoring the properties of the polymer matrix and fillers, the performance and application scope of CFRP can be further expanded [7,8,9]. Depending on the intended use, the composition of the polymer is selected to satisfy requirements related to manufacturing, functionality, durability and other critical design criteria [10,11]. Copper hydroxyapatite previously were used as an NIR light-absorbing additive in laser marking compounds and it can be a promising laser absorber in a 1030 nm region for epoxy-based composites to redistribute energy, reduce local overheating, and make material removal more controllable [12]. Also, calcium hydroxyapatite chemically related hydroxyapatite filler and can be used to partially replace copper hydroxyapatite and to evaluate the hydroxyapatite-type filler effect in composition on ablation efficiency and process stability [13].

In recent years, CFRP has been increasingly investigated for biomedical applications, including bone and joint replacement and CFRP-based implants for spinal surgery as an alternative to conventional titanium implants [8,14]. CFRP implants can generate significantly fewer artifacts in computed tomography (CT) and magnetic resonance imaging (MRI) compared to titanium, which can improve postoperative imaging and radiation therapy planning while maintaining comparable biomechanical and biocompatibility properties [8,15,16]. The radiolucency of CFRP enables more accurate radiation dose planning and delivery, and CFRP-based implants are generally considered potentially biocompatible and safe for use in the body without causing adverse reactions [17,18]. To further enhance implant performance, epoxy matrices can be modified with functional fillers such as copper hydroxyapatite (Cu-HAp), which improves thermal stability, mechanical strength and imparts antibacterial properties, and calcium hydroxyapatite (Ca-HAp), which is known for its bioactivity, non-toxicity and biocompatibility [19,20,21]. Ca-HAp-based materials can promote accelerated cell proliferation and spreading, rapid bioabsorption and regeneration when used as coatings for implants and for bone repair [22]. Such combinations of CFRP reinforcement with Cu-HAp and Ca-HAp fillers therefore offer a promising route toward mechanically robust, antibacterial and bioactive implant materials.

For these CFRP composites, the thermal stability and mechanical strength of the filler are key properties in many application areas, especially when laser processing is used for shaping or surface modification. In femtosecond laser processing, the interaction of materials with very different mechanical and thermal properties—carbon fibers and polymer/filler phases—strongly influences the quality and efficiency of material removal. CFRP systems containing Cu-HAp and Ca-HAp are also attractive because of their strong antibacterial effect against bacteria, such as Staphylococcus aureus and *Escherichia coli* [17,18], making them suitable for biomedical devices where a combination of mechanical strength and antibacterial performance is required. For such implant-oriented CFRP materials, laser processing offers the possibility to fabricate or adapt implants with minimal additional cost while maintaining high final quality [23,24].

Beyond bulk cutting, additional surface functionalities can be introduced by laser treatment of CFRP surfaces to generate controlled micro- and nano-scale topographies. Pre-designed carbon fiber weaving architectures and selective ablation or evaporation of the polymer filler can be used to open three-dimensional surface microstructures using either nanosecond [25] or femtosecond [26] laser pulses. In this way, surface properties can be tuned from hydrophilic [27] to hydrophobic [28], and bioactive coatings based on microstructured CFRP surfaces combined with hydroxyapatite additives can be created [29] to enhance initial cell adhesion and stimulate tissue growth. Because the melting temperature of carbon fibers (around 3500 °C) is much higher than that of typical epoxy/filler systems (200–350 °C) [30], there is considerable freedom to choose laser processing parameters to achieve desired evaporation/ablation quality and rate of the polymer filler and/or carbon fiber.

However, systematic studies of femtosecond laser ablation of CFRP containing Cu-HAp and Ca-HAp fillers, specifically with respect to ablation efficiency, optimal fluence ranges, cutting and milling rates, and the resulting surface quality relevant for implant applications, remain limited. In our previous work, we investigated femtosecond laser ablation of CFRP with a standard epoxy resin filler and showed that high-quality processing is achievable under appropriate conditions [31]. Building on that foundation, the present study focuses on femtosecond laser processing of CFRP with epoxy resin modified by different ratios and loadings of Cu-HAp and Ca-HAp additives. We quantify femtosecond laser ablation efficiency and optimal fluence ranges for CFRP based on epoxy modified with different Cu-HAp/Ca-HAp ratios and loadings, and directly compare these results with neat epoxy matrices containing the same additives. In addition, we demonstrate and quantify femtosecond laser cutting and milling performance (effective cutting speed, milling rate and heat-affected zone) of such Cu/Ca-HAp-modified CFRP, and discuss the implications for advanced, implant-oriented CFRP manufacturing. We focus exclusively on the laser processing behaviour of CFRP composites: no new biocompatibility, cytotoxicity, sterilization or mechanical validation experiments are performed, and biomedical aspects are discussed only in the context of previously reported data on CFRP and hydroxyapatite-based materials.

## 2. Materials and Methods

### 2.1. Optical Setup and Experimental Conditions

**Figure 1:**
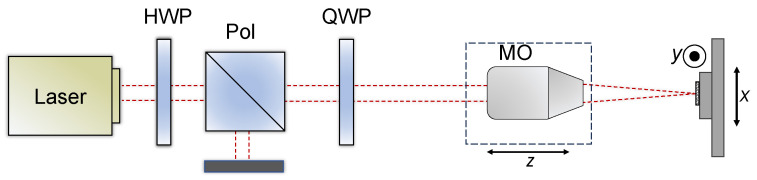
Optical setup for the fabrication of CFRP.

For the fabrication of CFRP, we employed the optical setup illustrated in Figure 1. For our process, a laser (Carbide, Light Conversion, Vilnius, Lithuania) that generates a Gaussian beam with a diameter of approximately 4.1 mm (at the $1/e^{2}$ level) and a wavelength of 1030 nm was used. A pulse duration of around 200 fs was used throughout these experiments. This laser can reach a maximum output of 6 W and is capable of operating at frequencies up to 200 kHz. Power control was achieved through an external attenuator comprising a half-waveplate (HWP) and a polarizer (Pol). The beam’s polarization was converted to circular by a quarter-waveplate (QWP), after which it was focused to a diameter of around 7.7 μm (at $1/e^{2}$ intensity level) using a 10X Mitutoyo Plan Apo NIR (0.26 NA) microscope objective mounted on a z-stage. The resulting focused beam had a Rayleigh length of roughly 37.5 μm. Proprietary CFRP or filler material sample was positioned on linear XY stages (Aerotech, Pittsburg, PA, USA). Samples were processed by hatching a 0.5 mm × 0.5 mm square area back and forth to ablate a cavity. A single scan was performed to form a single cavity. Spacing between the lines was set to 2.5 μm and pulse-to-pulse distance set to 0.6 μm, which corresponds to an overlap of 92%. Single pulse energy was varied from 0.5 μJ to 7 μJ and samples with different filler material were used in the experiment. Following laser treatment, samples were cleaned in distilled water within an ultrasonic bath, and the volume of the ablated squares was assessed with an optical profiler (Sensofar, Barcelona, Spain). We measured a profile of a single cavity and used the software’s measuring tool to determine the ablated volume. This tool allows the user to select a region of interest (ROI) and then it uses the area outside the selected region to determine the top plane and area inside the selected region to determine the bottom plane. The algorithm then calculates hole volume from the Z plane inside the ROI.

### 2.2. Sample Preparation

**Figure 2:**
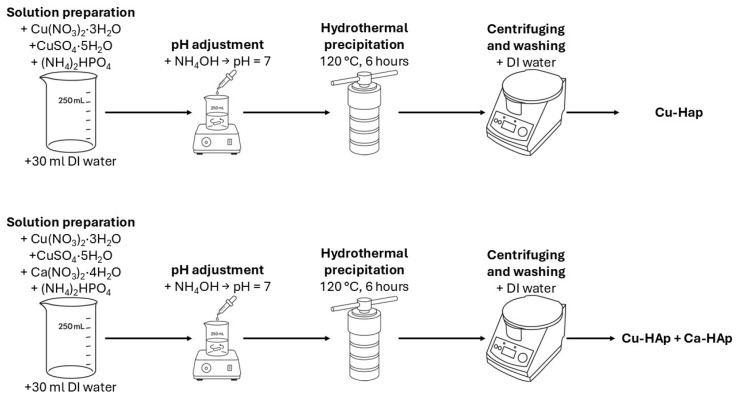
Sample preparation schematics.

Carbon fiber-reinforced plastic (CFRP) was obtained using CHS-EPOXY 619 epoxy resin and TELALIT 0600 hardener (Spolchemie, UstI nad Labem, Czech Republic). For clarity, sample preparation schematics are presented in Figure 2.

According to the component TDS, the resin had a viscosity of 400–900 mPa·s at 20 °C, epoxy index 5.9–6.5 mol/kg, and a density of 1.1 g·$cm^{-3}$ and EEW 155–170. The hardener had a viscosity of 80–120 mPa·s at 20 °C, amine number 450–500 mgKOH/g, and a density of 1.05 g·$cm^{-3}$ and HEW 62 g/mol. The resin-to-hardener mixing ratio recommended by the supplier was 40 phr, which was used throughout this work. A 3K carbon fabric with a density of 200 $g/m^{2}$ and a twill weave (Angeloni Group, Venice, Italy) was used as the reinforcing material.

Copper and calcium hydroxyapatite additives were obtained using a modified hydrothermal co-precipitation method based on the following papers [12,13]. First, a solution was prepared containing 0.02 mol of copper(II) nitrate trihydrate (Cu(NO_3_)_2_·3H_2_O) and 0.01 mol of copper(II) sulfate pentahydrate (CuSO_4_·5H_2_O) and Ca(NO_3_)_2_·4H_2_O with different molar ratios Cu and (Ca + Cu) was added, as well as 0.01 mol of diammonium phosphate (NH_4_)_2_HPO_4_, all dissolved in 30 mL of deionized (DI) water. The molar ratio of the prepared samples was selected to achieve the desired ratio of hydroxyapatite in the composition: copper hydroxyapatite (100%), copper hydroxyapatite (75%) + calcium hydroxyapatite (25%), copper hydroxyapatite (50%) + calcium hydroxyapatite (50%). The solution pH gradually increased to 7 using an aqueous ammonia solution while actively stirring with a magnetic stirrer. All reagents for the synthesis of hydroxyapatites were purchased from HLR Ukraine (Chemlaborreactiv LLC, Brovary, Ukraine). The resulting solution was then transferred to a PTFE cylinder in a hydrothermal reactor (BAOSHISHAN, Zhengzhou, China). The reactor was heated to 120 °C and maintained at this temperature for 6 h. The resulting product was filtered and purified using a centrifuge (CF-10, Daihan Scientific, Daejeon, Republic of Korea) and washed with deionized (DI) water. The pure hydroxyapatite obtained was dried at 60 °C for 2 h to produce the final product, which was used as an additive for CFRP.

To obtain laminated CFRP samples, the epoxy resin (CHS-EPOXY 619) was preheated to 50 °C to reduce viscosity. The hydroxyphosphate additive (Cu-HAp or Cu-HAp and Ca-HAp mixtures) was incorporated at 1 wt% and 5 wt% relative to the resin mass and dispersed using a high-speed disperser at 20,000 rpm for 5 min. The mixture was subsequently degassed under vacuum (5 kPa, 2 min) to remove entrapped air bubbles. The hardener (TELALIT 0600) was then added at a resin-to-hardener ratio of 40 phr, followed by gentle mixing with an overhead stirrer at 200 rpm for 5 min. The carbon fiber fabric was impregnated with epoxy resin using the hand layup method. Then, it was pressed under pressure to evacuate any remaining air pockets under low vacuum (50 kPa, 1 min). The resulting CFRP consisted of one ply with a fiber volume fraction of approximately 60%, estimated by the mass–density calculation method. Curing was carried out at room temperature for 72 h, followed by a post-curing heat treatment at 50 °C for 2 h to complete the cross-linking process.

For comparison experiments on the epoxy matrix without reinforcement, neat epoxy plates containing the same Cu-HAp or Cu-HAp/Ca-HAp additive loadings (1 wt% and 5 wt%) were prepared using the identical mixing/degassing protocol and cast into PTFE film-coated glass molds to obtain plates of 1 mm thickness. After curing and post-curing under the same conditions, both CFRP and neat epoxy specimens were cut into coupons of 3 × 5 cm for laser processing.

## 3. Experiments

### 3.1. Laser Ablation Efficiency Experiments

**Figure 3:**
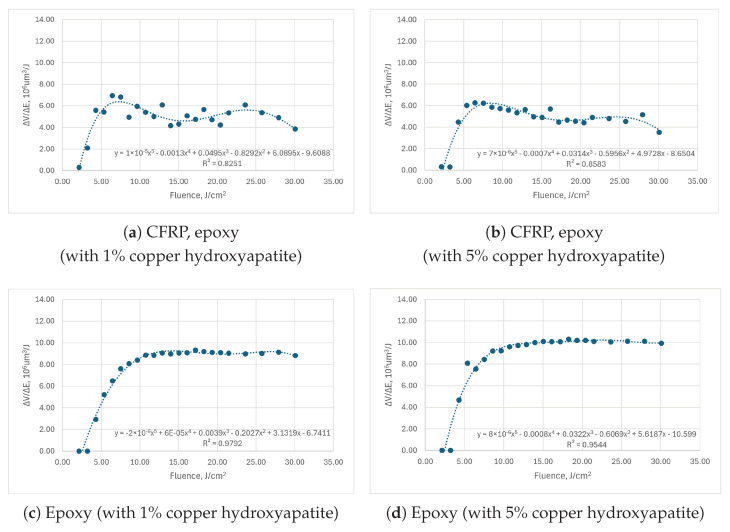
Comparison of CFRP and given mixture ratio of epoxy ablation efficiencies, to clarify the most efficient fluence threshold in order not to overcome it and not waste excess energy for processing. Also, it clarifies the differences and reasons of the ablation efficiencies for CFRP and copper hydroxyapatite nanoparticle percentages in mixtures of epoxy filler only. Ablation efficiencies of CFRP (**a**,**b**) and epoxy (**c**,**d**). Epoxy in (**a**,**c**) contains 1% copper hydroxyapatite. Epoxy in (**b**,**d**) contains 5% copper hydroxyapatite. Polynomial fit of the data is marked by dashed curve.

**Figure 4:**
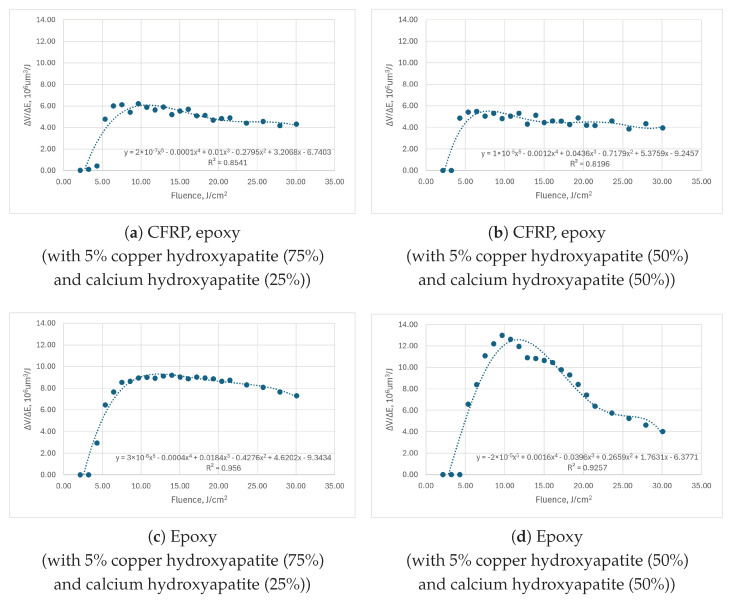
Comparison of CFRP and given mixture ratio of epoxy ablation efficiencies to clarify the most efficient fluence threshold in order to not overcome it and not waste excess energy for processing. Also, it clarifies the differences and reasons of ablation efficiencies for CFRP and copper hydroxyapatite with calcium hydroxyapatite in mixtures of epoxy filler only. Ablation efficiencies of CFRP (**a**,**b**) and epoxy (**c**,**d**). Epoxy in (**a**,**c**) contains a 5% mixture of copper hydroxyapatite (75%) and calcium hydroxyapatite (25%). Epoxy in (**b**,**d**) contains a 5% mixture of copper hydroxyapatite (50%) and calcium hydroxyapatite (50%). Polynomial fit of the data is marked by dashed curve.

**Figure 5:**
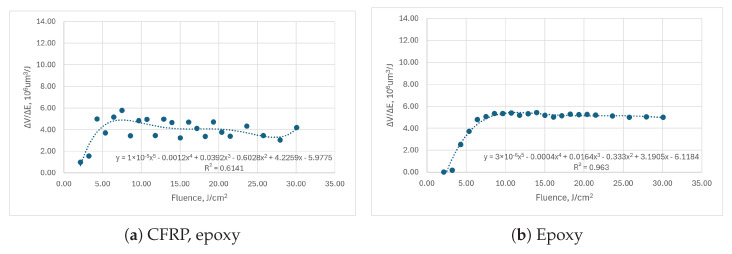
Comparison of CFRP and pure epoxy ablation efficiencies and clarification of the most efficient fluence threshold in order not overcome it and not waste excess energy for processing. Also, it clarifies the differences and reasons of ablation efficiencies for CFRP and pure epoxy filler only. Ablation efficiencies of (**a**) carbon fiber in pure epoxy resin, (**b**) pure epoxy resin. Polynomial fit of the data is marked by dashed curve.

In this section, we discuss laser ablation efficiencies of various samples. We focus on the comparison ablation differences of CFRP and epoxy resin alone. Both of these samples were processed as described in Section 2.1. Our aim is to investigate the influence of nano-particle additives, introduced into epoxy resin filler, to laser ablation efficiency. For each composition, four laser ablation cavities were fabricated at multiple positions distributed across the laminate surface, so that every fluence value in Figure 3, Figure 4 and Figure 5 is an average based on repeated measurements of several sites on a coupon. This approach ensures that the reported ablation efficiencies are representative of the material and not of a single local microstructural configuration. To smooth the experimental data and to extract approximate peak positions, we have used fifth-order polynomial fitting (plotted dashed lines) with equations and $R^{2}$ values provided as plot insets. The first results compare carbon fiber reinforced with epoxy resin containing different amounts of copper hydroxyapatite. CFRP ablation efficiency charts are presented in Figure 3a and Figure 3b, where copper hydroxyapatite content is 1% and 5%, respectively. In both of these cases, the maximum ablation efficiency was reached at around 6.4 $J/cm^{2}$ (single pulse energy 1.5 μJ). Measured energy-specific volume was slightly higher for sample with 1% salt content (6.95 μm^3^/μJ for 1% and 6.26 μm^3^/μJ for 5%). We next compared the ablation efficiencies of epoxy resin samples containing copper hydroxyapatite and no carbon fiber. In Figure 3c, a case where salt content is 1% is depicted, while in Figure 3d, it is 5%. The energy-specific volume increased to 9–10 μm^3^/μJ when increasing fluence to around 10 $J/cm^{2}$. Further fluence increase did not contribute to ablation efficiency in the tested energy range. Slightly higher energy-specific volume was reached on the 5% sample and the overall shape of both graphs is smoother compared to the CFRP ablation charts in Figure 3a,b. This shows that the introduction of carbon fiber into epoxy resin does influence the laser ablation process. When 6.4 $J/cm^{2}$ was selected, the ablation efficiencies of epoxy materials were 6.5 μm^3^/μJ (Figure 3c) and 7.5 μm^3^/μJ (Figure 3d). These values are close to the ablation efficiency of the CFRP at this fluence level. When higher fluences are selected, the ablation efficiencies of the CFRP material are lower than those of the epoxy samples. This indicates that the inclusion of carbon fiber limits the ablation process.

We next compared the laser processing of carbon fiber with epoxy resin containing a mixture of copper hydroxyapatite and calcium hydroxyapatite. Figure 4 depicts ablation efficiency measurements.

In Figure 4a, epoxy contained a 5 % mixture of copper hydroxyapatite (75%) and calcium hydroxyapatite (25%), while in Figure 4b, this 5% mixture had equal parts of copper hydroxyapatite (50%) and calcium hydroxyapatite (50%). There are only minor differences between the two plots. Slightly higher efficiency was achieved. Maximum ablation efficiency value (6.12 μm^3^/μJ) was achieved at the fluence of 7.52 $J/cm^{2}$ in the Figure 4a case, and in case Figure 4b, a maximum value of 5.47 μm^3^/μJ was achieved at 6.44 $J/cm^{2}$. Figure 4c,d show ablation efficiencies af epoxy containing a 5% mixture of copper hydroxyapatite (75%) and calcium hydroxyapatite (25%) (Figure 4c) and a 5% mixture of copper hydroxyapatite (50%) and calcium hydroxyapatite (50%) (Figure 4d), respectively. In Figure 4c, similar to the case before, the ablation efficiency steadily increases to around 9 μm^3^/μJ with increasing fluence to around 10 $J/cm^{2}$. Very little change was registered with further fluence increase to around 20 $J/cm^{2}$, after this value ablation efficiency started to drop. In Figure 4d, ablation efficiency rises to around 13 μm^3^/μJ when increasing fluence to 10 $J/cm^{2}$ and then it drops with further fluence increase. The ablation process generated a certain amount of debris that shields further ablation of specimens and we observed a decrease in ablation efficiency, as shown in Figure 4c,d. Since the physical properties of carbon fiber and polymer filler are quite different, we found different ablation efficiency dependencies for CFRP and pure polymer filler. Both cases of filler additive mixture ratios (Figure 3 and Figure 4) show lower CFRP ablation efficiency and are influenced by carbon fiber’s own response to femtosecond pulse. Carbon fiber is much more resistant to femtosecond pulse ablation compared to polymer filler, so the ablation efficiency numbers for CFRP are lower in laser fluence dependences.

Lastly, Figure 5 shows the ablation efficiencies of CFRP in epoxy with no additives (Figure 5a) and pure epoxy with no additives (Figure 5b). In both cases, maximum ablation efficiency is close to 6 $J/cm^{2}$. Overall, for CFRP, it is slightly lower than efficiencies where carbon fiber was in epoxies containing hydroxyapatites. For pure epoxy, it is noticeably lower than for epoxies with hydroxyapatites. This indicates that hydroxyapatite inclusion can enhance ablation efficiency. In general, the individual points on the graphs related to CFRP ablation seem more noisy than in the case of epoxy ablation. The squares were ablated on a different position in a sample; therefore, the density of fibers might not be constant. Fibers are more densely packed in the middle, while on the surface, more volume is filled with epoxy, and fiber density seems to vary more. This is clearly visible in further sample inspection after cutting it, which is described in the next section.

### 3.2. CFRP Cutting and Milling by Ablation

We next tried to increase the processing speed by modifying the beam delivery system. Instead of XY positioning stages, we used a galvanometer scanner together with an f-theta lens (f = 100 mm). This configuration produced a beam spot diameter of roughly 24 μm on the material surface. Additionally, the focusing depth achieved with the f-theta lens is approximately ten times greater than that of the earlier setup. For further sample processing, we kept pulse duration the same as in previous experiments, and proportionally, scale pulse energy, pulse-to-pulse and line-to-line distances were also scaled. The laser was operated at 200 kHz at 3.4 W of power. Under these conditions, the single pulse energy was 17 μJ, which corresponds to a fluence of 7.5 $J/cm^{2}$. To maintain the same line-to-line and pulse-to-pulse overlap ratio, the scanning speed was set to 374 mm/s, and line spacing was set to 15.4 μm.

**Figure 6:**
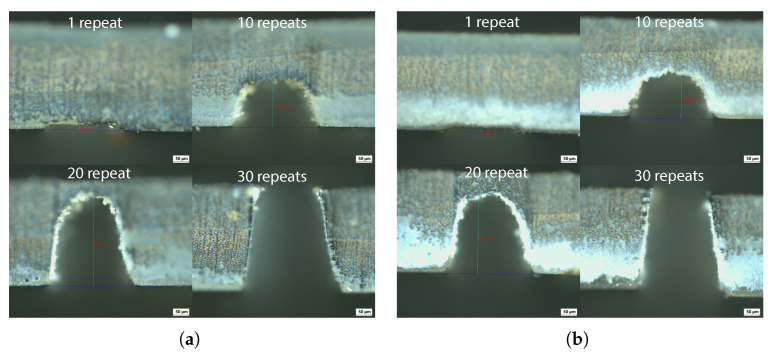
Cross sections of cutting streets after performing a different number of scans on CFRP. In (**a**) epoxy contains a 1% mixture of copper hydroxyapatite (75%) and calcium hydroxyapatite (25%). In (**b**) epoxy contains a 1% mixture of copper hydroxyapatite (50%) and calcium hydroxyapatite (50%).

To evaluate the rate of laser processing for CFRP containing certain compounds, we performed cutting experiments using the described conditions. For the conceivable implant manufacturing applications, it is crucial to know the possibility of forming the shape of exchangeable bones elements with reasonable processing time and surface quality. We have performed the simplest process of a cutting procedure that can be easily extrapolated to a milling process with the evaluation of milling rate and processing item surface quality. A 250 μm wide channel was ablated with an increasing number of scans until a full cut of the sample was achieved. In Figure 6, cutting experiment results are shown for two different 400 μm thick CFRP samples: in the first one (Figure 6a), epoxy contains a 1% mixture of copper hydroxyapatite (75%) and calcium hydroxyapatite (25%), and in the second one (Figure 6b), it contains a 1% mixture of copper hydroxyapatite (50%) and calcium hydroxyapatite (50%).

For both samples, around 30 scanning repeats were needed to achieve full cut. Under these conditions, the effective cutting speed for the 400 μm thick CFRP sample was around 0.7 mm/s. Considering this for the milling process, 1 cubic centimeter of material could be removed in around 4 h of ablation. This is a quite acceptable milling volume removal rate for setup utilizing a low-power femtosecond laser. To further enhance the milling rate, it is possible to apply a higher power femtosecond laser and advanced beam delivery setup. Such an improvement could shorten the process time significantly and elevate the method to be applicable in industrial applications.

**Figure 7:**
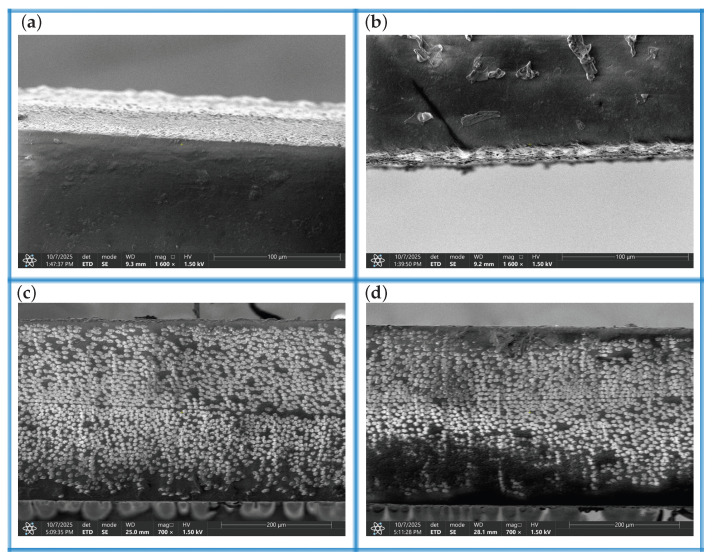
SEM pictures of CFRP. The epoxy in (**a**,**c**) contains a 1% mixture of copper hydroxyapatite (75%) and calcium hydroxyapatite (25%). The epoxy in (**b**,**d**) contains a 1% mixture of copper hydroxyapatite (50%) and calcium hydroxyapatite (50%). The surface of the sample edge is depicted in (**a**,**c**), and a cross section of the sample edge is depicted in (**c**,**d**).

We further evaluate the ablation quality by inspecting the samples using SEM an and optical profilometer. SEM (Thermo Fisher Scientific, Waltham, MA, USA) images of sample surfaces close to the edge are presented in Figure 7a,b and cross sections of the cut are shown in Figure 7c,d.

**Figure 8:**
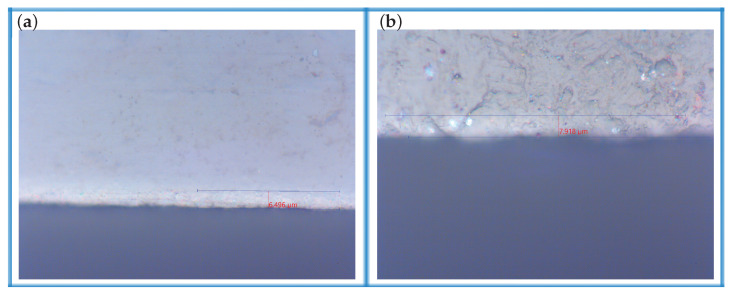
Optical microscope pictures of the surface of CFRP after cutting. The epoxy in (**a**) contains a 1% mixture of copper hydroxyapatite (75%) and calcium hydroxyapatite (25%). The epoxy in (**b**) contains a 1% mixture of copper hydroxyapatite (50%) and calcium hydroxyapatite (50%).

In Figure 7a,c, CFRP epoxy contains a 1% mixture of copper hydroxyapatite (75%) and Calcium hydroxyapatite (25%), while in Figure 7b,d, the epoxy contains a 1% mixture of copper hydroxyapatite (50%) and calcium hydroxyapatite (50%). In both samples, very little change is noticeable on the surface close to the edge of the cut (Figure 7a,b). We determined the heat-affected zone (HAZ) after ablation by measuring the discoloration of the material starting from the sample edge (see Figure 8). HAZ on the sample after cutting is negligible (around 8 μm). Low HAZ width shows the good sustainability of these CFRP materials. This is a good result from the perspective of the femtosecond laser processing of this material. The method let us avoid significant modification on the edge of CFRP parts. Cross sections of the cut (Figure 7c,d) show that the fibers in the material are more densley packed around the center of the sample. Even though individual fibers can be detected under SEM, the overall surface roughness (Sa) of the edge is quite low. We used an optical profilometer to measure the Sa (the arithmetic mean height of the surface) of the sample edge presented in Figure 7c to be around 1.3 μm, and in the case presented in Figure 7d, the Sa is around 0.9 μm. Such edge quality is acceptable for CFRP parts manufactured for different applications. Surface measurements were performed using an optical profilometer and the measured cross section length was 850 μm.

## 4. Discussion

CFRP is a compound material with very different physical properties of the main components—carbon fiber and epoxy resin filler. Laser processing of CFRP in some publications [27,32,33] is demonstrating the surface selective texturing to regulate surface bonding performance or wettability to provide desired properties from super-hydrophilic (contact angle 0°) up to super-hydrophobic (contact angle 175°). It achieves the target necessary to obtain micron-scale resolution of surface texturing. In publication [23], the authors obey the internal structure of carbon fiber in CFRP to achieve the desired properties using various laser pulse widths. Composites can be made of materials with contrasting properties. In CFRP, femtosecond processing may selectively modify the matrix while leaving reinforcements intact. We are working with the femtosecond laser processing of CFRP, targeting to control the ablation process to achieve uniform removal of the material. The results are presented and compared with pure epoxy resin CFRP filler and mixtures with nanoparticle additives that change laser pulse energy distribution in material and increase ablation uniformity. Well-known so-called material “cold ablation” in femtosecond processing is applicable to CFRP compound materials mixed with nanoparticle additives and enables the processing of different constituents with minimal thermal damage. As shown in our experimental results, the most suitable ablation efficiency of 6–7 μm^3^/μJ is achieved at laser fluence in the range of 6–8 $J/cm^{2}$, which can be used to optimize process parameters, avoid excess laser energy application, increase the laser process rate and evaluate the limits for enhancing manufacturing throughput. Our investigation does not include quantitative results of carbon fiber ablation without any epoxy compounds. Such a comparison could in principle provide further insight into the processing differences. However, under our conditions, femtosecond laser ablation cuts individual fibers and destroys the weaving of the sample, so that the material no longer forms a continuous surface and the resulting voids cannot be described as well-defined cavities for volumetric profilometry. A meaningful, quantitative study of “pure carbon fiber” ablation would therefore require a different sample geometry and diagnostic approach, and is left for future work.

Our experimental findings are consistent with and extend several recent studies on ultrafast laser processing of CFRP and related composites. The lower ablation efficiencies observed in CFRP compared with neat epoxy at the same fluence (Figure 3, Figure 4 and Figure 5) can be traced to three coupled microstructural mechanisms that are directly reflected in our data. First, anisotropic heat transport along the carbon fibers redistributes energy away from the irradiated matrix region and reduces the local peak temperature in the epoxy; this leads to lower material removal per unit energy in CFRP than in neat epoxy at identical fluence. This effect is consistent with the fact that, at 10 $J/cm^{2}$, the energy-specific volume of neat epoxy with Cu-HAp is about 9–10 μm^3^/μJ (1 wt%) and 9–13 μm^3^/μJ (5 wt%), whereas CFRP with the same additives reaches only 6–7 μm^3^/μJ at its optimum fluence window of 6.4–7.5 $J/cm^{2}$. Second, spatial variability in fiber packing modifies the local effective ablation threshold. Cross-sectional images (Figure 7c,d) show that fibers are more densely packed near the laminate mid-plane, while regions closer to the surface contain a larger epoxy fraction and more variable packing density. This heterogeneity agrees with the observed scatter of CFRP ablation data and with the tendency for slightly reduced peak energy-specific volumes in CFRP compared with neat epoxy, because areas with higher local fiber volume fraction require more energy to remove the same volume of material. The increased point-to-point scatter and slightly lower peaks in the CFRP curves relative to the smoother epoxy curves (Figure 3 and Figure 4) could indicate that the response is sensitive to the local fiber structure. Third, additive-enabled optical absorption is expected to play a role: Cu-HAp/Ca-HAp particulates have been reported to act as near-IR absorbers [12,13], and within the epoxy, they would redistribute deposited energy at the microscale. Based on these literature data and on the systematic differences in ablation--efficiency curves between epoxy with and without Cu/Ca-HAp (Figure 3, Figure 4 and Figure 5), we qualitatively attribute part of the enhanced matrix removal efficiency to increased absorption at 1030 nm, while carbon fibers remain comparatively more resilient [34] (the optical properties of the specific composites studied here were not measured directly, so this interpretation should be viewed as qualitative). At higher fluence, debris/particle shielding and fiber-dominated scattering emerge: resin removal exposes fiber bundles that scatter the incident beam and trap/deposit ejecta, both of which reduce incremental removal per additional energy input (efficiency roll-off). Similar shielding-induced saturation is widely reported in ultrafast ablation and is consistent with our efficiency peak near 6–8 $J/cm^{2}$ followed by a smooth decline. The combination of these mechanisms explains both the reduced peak ablation efficiency and the noisier fluence dependence observed for CFRP compared with neat epoxy.

The comparison with recent microstructure-aware studies is as follows. Konishi and Yan [34] showed that fiber orientation strongly modulates femtosecond micromachining due to preferential heat conduction along fibers; surfaces scanned perpendicular to fiber direction became uneven—a trend we also see in the increased scatter of CFRP efficiencies versus neat epoxy (orientation-averaged in our 0.5 × 0.5 mm hatches). Zuo et al. [27] leveraged selective matrix removal and controlled fiber exposure to engineer wettability and chemistry changes (sp^2^ → sp^3^ in fibers), underscoring that, at low fluence, the matrix is preferentially ablated—consistent with our higher matrix-only efficiencies and the lower CFRP efficiencies at the same fluence. Wu et al. [35] reported negligible HAZ for femtosecond versus visible HAZ for picosecond processing of CFRP, in line with our ~8 µm edge discoloration/HAZ and sub 2 µm edge roughness.) Li et al. [32] quantified scan-angle-dependent removal thresholds and identified an optimal femtosecond energy density window (≈12–13 J cm^−2^) for selective texturing; our efficiency peak at ~6–8 J cm^−2^ lies lower because our metric emphasizes volume per energy during cavity milling rather than onset thresholds for surface texturing. In drilling, Jiang et al. [24] achieved cylindrical holes with HAZ < 10 µm by spiral femtosecond trepanning; our channel-cut tests (400 µm thick, full separation in ~30 passes) complement those results by quantifying bulk milling rate under scanner delivery. Finally, finite-element and mixed-scale models that embed anisotropic fiber conductivity reproduce elliptical heat footprints and angle-dependent kerf geometry under pulsed irradiation, mirroring our observation that CFRP efficiency curves are “noisier” and slightly lower than neat epoxy due to microstructure-dictated heat spreading and debris dynamics. Taken together, this agreement with recent microstructure-aware experiments and modeling confirms that the efficiency trends and HAZ characteristics observed in our Cu/Ca-HAp-modified CFRP lie within the same physical framework as reported for other CFRP systems and ultrafast laser conditions.

With respect to filled polymer composites, our finding that Cu-/Ca-HAp additives slightly raise ablation efficiency in CFRP while modifying surface quality in neat epoxy is consistent with reports that near-IR absorbing copper hydroxyphosphate (libethenite) increases matrix absorption at 1 μm, thereby redistributing deposited energy at the micro-scale.

The outlook for the future studies could be microstructure-informed modeling to complement experiments. A practical route to predictive process design is a two-stage multi-scale model. Stage 1 (photon transport): compute heterogeneous energy deposition within a 3D voxelized domain (fibers, epoxy, Cu-/Ca-HAp) using a Monte Carlo radiative-transfer solver (MCML/MCmatlab-style or GPU-accelerated variants) with wavelength-specific optical properties (absorption/scattering) for each phase. This captures local hot-spots at absorber particulates and fiber-induced scattering/anisotropy. Stage 2 (thermal and removal): couple the absorbed energy to a two-temperature/thermal model augmented for composites: anisotropic thermal conductivity tensors for fibers, isotropic resin properties, and an incubation/accumulation term to account for pulse-number-dependent thresholds under scanning. A simple, data-driven ablation law (e.g., depth increment when local fluence exceeds a phase-specific threshold) is then calibrated against measured ablation volumes and HAZ metrics. Use cases: (a) scan-angle optimization relative to local fiber direction to minimize efficiency loss from axial heat spreading; (b) additive loading/dispersion studies to balance matrix absorption gains versus debris shielding; (c) repetition-rate/overlap tuning to exploit incubation without excessive heat accumulation. We plan to validate the model by (i) acquiring voxelized microstructures from SEM cross sections/μCT, (ii) measuring spot-wise thresholds and efficiency versus angle, and (iii) comparing predicted versus measured cavity volumes and HAZ across the 6–8 J cm^−2^ efficiency window identified here.

CFRP with special filler compounds and ratio percentages as a mixture of copper hydroxyapatite and calcium hydroxyapatite effective in terms of biocompatibility, having a strong antibacterial effect [16,19,22], are promising materials for the implant industry. The investigation shows the possibility to process CFRP with high processed surface quality (roughness < 2 μm) and acceptable milling rate of about one cubic centimeter ablation within around 4 h using even a low-power (6 W) femtosecond laser. These findings provide new application prospects for femtosecond lasers.

## 5. Conclusions

In this work, we investigated the femtosecond laser ablation of carbon fiber-reinforced polymer (CFRP) based on epoxy resin modified with different ratios and loadings of copper hydroxyapatite (Cu-HAp) and calcium hydroxyapatite (Ca-HAp) additives. For CFRP samples containing 1 and 5 wt% Cu-HAp, the maximum ablation efficiency was obtained at fluences in the range of 6.4–7.5 $J/cm^{2}$, with energy-specific volumes of about 6–7 μm^3^/μJ, while neat epoxy with the same additives reached higher energy-specific volumes of approximately 9–13 μm^3^/μJ at 10 $J/cm^{2}$. The inclusion of hydroxyapatites enhanced ablation efficiency in epoxy, whereas CFRP showed lower efficiencies at the same fluence due to the influence of carbon fibers, anisotropic heat transport and debris shielding.

Cutting experiments using a galvanometer scanner and 6 W femtosecond laser demonstrated that 400 μm thick CFRP samples could be fully separated in about 30 passes, corresponding to an effective cutting speed of around 0.7 mm/s and an estimated milling rate of around 1 $cm^{2}$ in around 4 h, with a negligible heat-affected zone (around 8 μm) and edge roughness on the order of 1 μm, which is acceptable for many CFRP component applications. Considering the simultaneously achievable surface quality, processing rate and the known bioactive and antibacterial potential of Cu/Ca-HAp-modified CFRP, these results therefore not only provide processing windows for tested materials, but also confirm the realistic prospects of femtosecond laser processing for advanced implant-oriented CFRP manufacturing.

Finally, we note that no direct measurements of absorption, reflection or scattering were performed on the investigated composites at 1030 nm. The optical mechanism discussion is therefore qualitative and supported by the literature data on Cu/Ca-containing hydroxyapatite systems rather than by new spectroscopic measurements. We also note that this work is limited to the characterization of laser ablation efficiency, cutting and milling performance and resulting surface quality. No biocompatibility, cytotoxicity, sterilization, mechanical strength or fatigue tests were carried out on the investigated composites, and any biomedical implications are therefore to be understood as an outlook based on previously published properties of CFRP and Cu/Ca-HAp systems.

## Data Availability

Data are available at http://doi.org/10.17632/sdz48p9j5m.1.

## Abbreviations

The following abbreviations are used in this manuscript:

CFRP	Carbon Fiber-Reinforced Plastic
HAZ	Heat-Affected Zone
Ca-HAp	Calcium hydroxyapatite
Cu-HAp	Copper hydroxyapatite
